# Vaccine Containing the Three Allelic Variants of the *Plasmodium vivax* Circumsporozoite Antigen Induces Protection in Mice after Challenge with a Transgenic Rodent Malaria Parasite

**DOI:** 10.3389/fimmu.2017.01275

**Published:** 2017-10-11

**Authors:** Alba Marina Gimenez, Luciana Chagas Lima, Katia Sanches Françoso, Priscila M. A. Denapoli, Raquel Panatieri, Daniel Y. Bargieri, Jean-Michel Thiberge, Chiara Andolina, Francois Nosten, Laurent Renia, Ruth S. Nussenzweig, Victor Nussenzweig, Rogerio Amino, Mauricio M. Rodrigues, Irene S. Soares

**Affiliations:** ^1^Department of Microbiology, Immunology and Parasitology, Center of Cellular and Molecular Therapy (CTCMol), Federal University of São Paulo, São Paulo, Brazil; ^2^Department of Clinical and Toxicological Analyses, School of Pharmaceutical Sciences, University of Sao Paulo, São Paulo, Brazil; ^3^Unit of Malaria Infection and Immunity, Institut Pasteur, Paris, France; ^4^Department of Parasitology, University of São Paulo, São Paulo, Brazil; ^5^Shoklo Malaria Research Unit, Mahidol-Oxford Tropical Medicine Research Unit, Faculty of Tropical Medicine, Mahidol University, Mae Sot, Thailand; ^6^Centre for Tropical Medicine and Global Health, Nuffield Department of Medicine Research Building, University of Oxford, Oxford, United Kingdom; ^7^Singapore Immunology Network, Biopolis, Agency for Science Technology and Research, Singapore, Singapore; ^8^New York University School of Medicine, New York, NY, United States

**Keywords:** malaria, *Plasmodium vivax*, recombinant vaccine, circumsporozoite protein, prime-boost regimens

## Abstract

*Plasmodium vivax* is the most common species that cause malaria outside of the African continent. The development of an efficacious vaccine would contribute greatly to control malaria. Recently, using bacterial and adenoviral recombinant proteins based on the *P. vivax* circumsporozoite protein (CSP), we demonstrated the possibility of eliciting strong antibody-mediated immune responses to each of the three allelic forms of *P. vivax* CSP (PvCSP). In the present study, recombinant proteins representing the PvCSP alleles (VK210, VK247, and *P. vivax*-like), as well as a hybrid polypeptide, named PvCSP-All epitopes, were generated. This hybrid containing the conserved C-terminal of the PvCSP and the three variant repeat domains in tandem were successfully produced in the yeast *Pichia pastoris*. After purification and biochemical characterization, they were used for the experimental immunization of C57BL/6 mice in a vaccine formulation containing the adjuvant Poly(I:C). Immunization with a recombinant protein expressing all three different allelic forms in fusion elicited high IgG antibody titers reacting with all three different allelic variants of PvCSP. The antibodies targeted both the C-terminal and repeat domains of PvCSP and recognized the native protein on the surface of *P. vivax* sporozoites. More importantly, mice that received the vaccine formulation were protected after challenge with chimeric *Plasmodium berghei* sporozoites expressing CSP repeats of *P. vivax* sporozoites (Pb/PvVK210). Our results suggest that it is possible to elicit protective immunity against one of the most common PvCSP alleles using soluble recombinant proteins expressed by *P. pastoris*. These recombinant proteins are promising candidates for clinical trials aiming to develop a multiallele vaccine against *P. vivax* malaria.

## Introduction

Morbidity due to malaria is prevalent worldwide and is reflected by millions of cases every year, which are almost entirely attributable to *Plasmodium falciparum* and *Plasmodium vivax* parasite species infection (1). *P. vivax* is the most geographically widespread species of human malaria, predominating in the regions of North and South America, South and Southeast Asia, Western Pacific, and Eastern Mediterranean; according to the WHO 2015 World Malaria Report, this malarial species was responsible for 13.8 million cases worldwide in 2015 (1). Complicated malaria and deaths due to *P. vivax* malaria have been reported in different endemic regions (2). Despite the studies on *P. vivax* malaria being neglected in the last few decades (3–5), currently, there is a consensus that studies aiming to control and eliminate this important tropical disease are of high priority (6). In this context, the development of a *P. vivax* vaccine would represent a great advancement in malaria control strategies. Unfortunately, only four clinical trials based on the *P. vivax* antigens (PvDBP, ScPvs25, N-R&C peptides and VMP001) have been completed according to the NIH website.^1^

The circumsporozoite protein (CSP) is the most abundant component of sporozoite surface and is involved in the initial stages of invasion of mammalian host hepatocytes [reviewed in Ref. (7, 8)]. As the first protein associated with an effective protection against malaria (9, 10), the majority of trials in experimental animals and humans are based on the CSP. This protein is an important target for antibodies and CD4^+^ and CD8^+^ T cells that can eliminate the preerythrocytic stages of the parasite (11). RTS,S, the most advanced vaccine so far, directed to the *P. falciparum* CSP, now named as Mosquirix™, is currently scheduled to begin a pilot phase in sub-Saharan Africa in 2018, according to WHO program (12). This formulation uses the hepatitis B surface antigen as a matrix for the conserved C-terminal portion and central repeat domain of CSP combined to powerful adjuvant systems (AS), which is a liposomal suspension (AS01) presentation of the immunostimulants monophosphoryl lipid A (MPL) and saponin purified from *Quillaja saponaria* (QS21). The Phase III efficacy of RTS,S against all malaria episodes between children and young infants residents of African endemic areas ranges from 28% (three-dose group) to 36% (four-dose group) (13). These promising RTS,S results justify investment in the CSP as a target for *P. vivax* vaccines.

As described for the CSPs of other species of *Plasmodium*, the primary structure of *P. vivax* CSP (PvCSP) has three major defined domains. The central repeat domain is flanked by highly conserved regions, the N- and C-terminal domains. All three domains can be targets of specific antibodies. However, only antibodies directed against the central repeat domain of PvCSP have been associated to protective efficacy against *P. vivax* in *Aotus nancymaae* monkeys (14). Sequencing of the genes encoding the CSPs of different strains of *P. vivax* uncovered the presence of three different alleles at the central repeat domain. These alleles have been described in different parts of the world (15–19). This strain diversity adds complexity for the development of a multiallelic vaccine against *P. vivax* malaria. The CSPs designated VK210, VK247, and *P. vivax*-like are almost identical in their N- and C-terminal domains, but differ in the central repeat region.

*P. vivax* CSP-derived antigens have been combined into multivalent formulations or chimeric synthetic molecules in attempts to obtain protective immunity against *P. vivax*. Recombinant PvCSP-derived proteins expressed in *Escherichia coli* and yeast were tested as vaccines with very limited success (20) and thus were not pursued further. Peptides based on the N-terminal region, central repeats, and C-terminal region of PvCSP-VK210 formulated in Montanide ISA 720 or Montanide ISA 51 adjuvants were immunogenic in BALB/c mice, *Aotus* monkeys (21), and healthy human volunteers (22). Recently, a PvCSP-based vaccine named Rv21 was demonstrated to be highly protective against challenge in rodent models to malaria (23). However, these vaccines did not consider the three allelic variants of PvCSP.

The most advanced recombinant protein formulation for *P. vivax* is the vaccine VMP001 (vivax malaria protein 001) (24). VMP001, obtained in bacteria *E. coli*, merges the central variant epitopes of VK210 and VK247 flanked by the amino- and carboxy-terminal regions of PvCSP, and was immunogenic in C57BL/6 (25), *Rhesus* monkeys (26), and human naive volunteers (27). The clinical trial results from Phase I/IIa showed VMP001 to be immunogenic, inducing humoral and cellular immune responses to the vaccine antigen. A significant delay in time to parasitemia was seen in 59% of vaccinated subjects compared to the control group. However, vaccination did not induce sterile protection (27). Immunogenicity to PvCSP was also achieved by our group, with *E. coli* constructs fusing all of the three variants, in C57BL/6 mice (28, 29). In these studies, the induced antibodies against the PvCSP chimeric constructs were able to recognize epitopes from each of the variants (VK210, VK247, and *P. vivax*-like) inserted in the central region (28, 29). However, the recombinant PvCSP used in these studies were expressed using prokaryotic systems as insoluble proteins, complicating large scale production. Thus, antigen expression as soluble secreted proteins using eukaryotic systems such as *Pichia pastoris* may represent a long-term advantage in an effort to solve this problem.

Based on these findings, we generated three new recombinant proteins based on the central regions of these variants, that contain immunodominant epitopes for B cells, and the conserved C-terminal region of PvCSP. Additionally, we generated a fourth recombinant protein, which contains the C-terminal region and epitopes from the three *P. vivax* CSP alleles fused as a single polypeptide, called yPvCSP-All epitopes. All recombinant proteins were successfully produced as soluble secreted proteins in the yeast *P. pastoris*. The present study describes the immunogenicity of the different formulations and analysis for protection against the PvCSP-VK210 allele.

## Materials and Methods

### Ethics Statement

This study was performed in strict accordance with the recommendations in the Guide for the Care and Use of Laboratory Animals of the Brazilian National Council for the Control of Animal Experimentation (CONCEA).^2^ The protocol was approved by the Committees on the Ethics of Animal Experiments of the Faculty of Pharmaceutical Sciences of University of São Paulo, Brazil (CEUA No. 362/2012), and the Institutional Animal Care and Use Committee at the Federal University of Sao Paulo (CEP No. 0172/12 and CEUA No. 1463171214). The study with human blood obtained from infected Thai patients was approved by Oxford Tropical Research Ethics Committee (reference OX28-09).

### Recombinant Proteins Expressed in *P. pastoris* Yeast

#### Genetic Design and Construction of yPvCSP Recombinant Proteins

**Figure 1:**
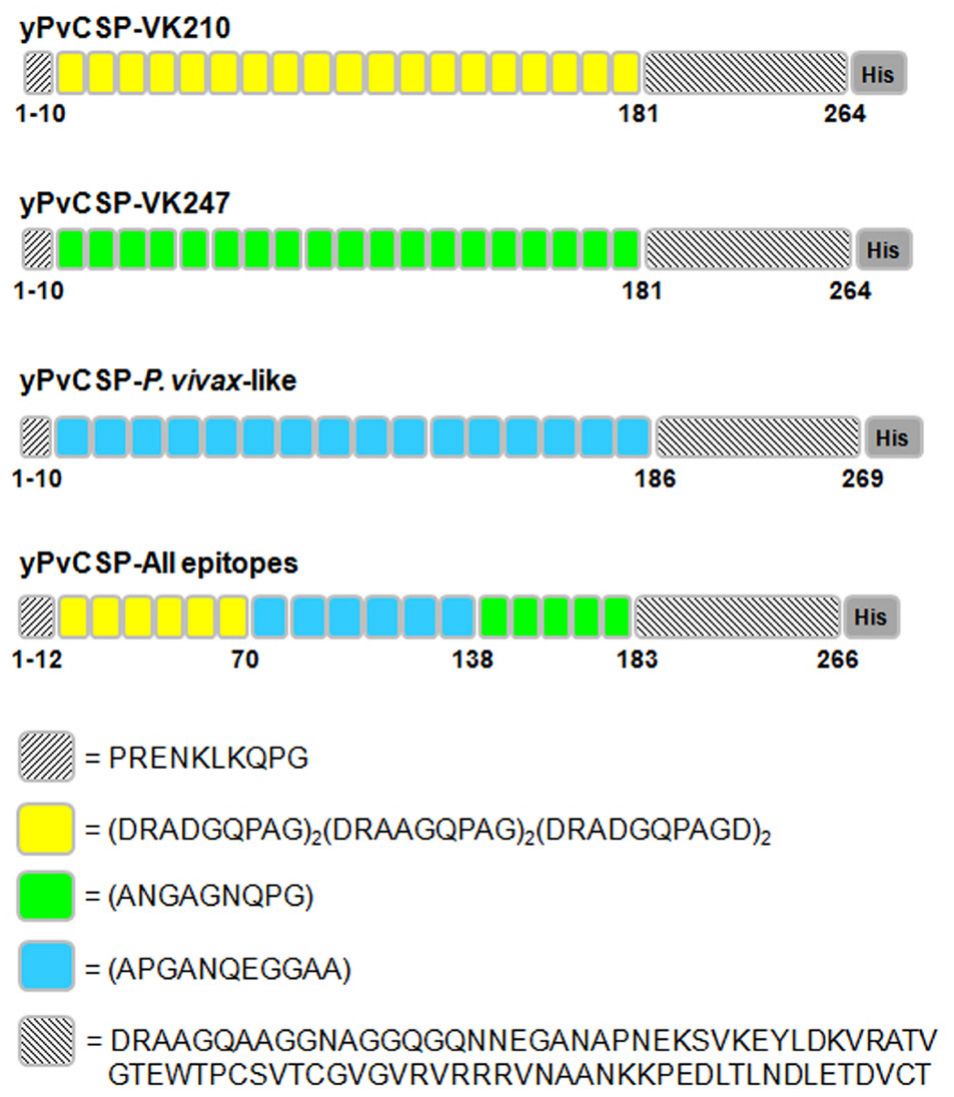
Schematic representation of the *Plasmodium vivax* circumsporozoite protein (CSP) recombinant antigens. The individual recombinant proteins have the specific individual repeats [yPvCSP-VK210 (*n* = 19), yPvCSP-VK247 (*n* = 19), or yPvCSP-*P. vivax*-like (*n* = 16)] and the C-terminal (CT) region. For each repeat sequence, the amino acids positions and the histidine tag are indicated. The classic repeat sequence VK210 is represented in yellow, VK247 in green, and *P. vivax*-like in blue. The yPvCSP-All epitopes merges the repeats of all three allelic variants describe above (*n* = 6/6/5 repeat sequences of VK210/*P. vivax*-like/VK247, respectively) and the CT region.

Synthetic genes encoding the recombinant proteins yPvCSP-VK210, yPvCSP-VK247, yPvCSP-*P. vivax*-like, and yPvCSP-All epitopes were synthetized by GenScript USA, Inc. (Piscataway, NJ, USA) using codon optimization to improve expression in *P. pastoris*. Figure 1 shows a schematic representation of these recombinant proteins. The synthetic genes were cloned into the pUC57 vector and were subsequently subcloned into the *P. pastoris* expression vector pPIC9K (Invitrogen, Carlsbad, CA, USA). Amplified plasmids were linearized with *Sal*I and transformed into the GS115 strain (his4^−^) of *P. pastoris* by electroporation. Clones transformed with plasmid *pPIC9K-PvCSP-VK210, pPIC9K-PvCSP-VK247, pPIC9K-PvCSP-P. vivax-like*, or *pPIC9K-PvCSP-All epitopes* were screened for high copy-number integration by G418 selection, according to the manufacturer’s instructions (*Pichia* Expression Kit, Invitrogen).

#### Expression and Purification of Recombinant Proteins

Clones with a Mut^+^ phenotype that were secreting high levels of each recombinant protein were selected. The induction of protein expression was performed as described, with some modifications (30). A Mut^+^ transformant was initially grown overnight in 200 mL of BMGY medium (1% w/v yeast extract, 2% w/v peptone, 1.34% w/v yeast nitrogen base without amino acids, 4 × 10^−5^% w/v biotin, 1% w/v glycerol, and 0.1 M potassium phosphate, pH 6.0) at 28–30°C with vigorous shaking. The cells were harvested, resuspended in 1 L BMMY (BMGY with glycerol replaced with 0.5% v/v methanol), and incubated again for 72 h. Methanol was added every 24 h to a final concentration of 1% v/v. After induction for 72 h, the cells were removed by centrifugation and the culture supernatant was clarified by filtration with a 0.45-µm membrane (Merck Millipore, MA, USA). The supernatant was applied to a HisTrap FF column coupled to a FPLC ÄKTA prime plus (GE Healthcare, Chicago, IL, USA), which was previously equilibrated with 20 mM sodium phosphate/0.5 M NaCl, pH 8.0. Bound proteins were eluted with a 0–500 mM imidazole (Sigma-Aldrich, St. Louis, MO, USA) gradient in 20 mM sodium phosphate/0.5 M NaCl, pH 8.0. Fractions containing protein were detected by SDS-PAGE and Coomassie blue staining, pooled, and used in a second-purification step by anionic exchange chromatography using a Resource Q column coupled to a FPLC ÄKTA prime plus (GE Healthcare, Chicago, IL, USA). The protein was eluted using a 0–1 M NaCl linear gradient in 20 mM Tris–HCl, pH 8.0, and analyzed by SDS-PAGE. The peaks corresponding to each recombinant protein that had a high degree of purity were collected and dialyzed against phosphate-buffered saline (PBS). The protein concentration was determined by the Bradford method (BioRad, Hercules, CA, USA) using bovine serum albumin (BSA; Sigma-Aldrich, St. Louis, CA, USA) as a standard.

### Characterization of Recombinant yPvCSP

#### Immunoblotting

For immunologic characterization, the recombinant proteins were fractionated by 12% SDS-PAGE under reducing conditions and were transferred from the gel to nitrocellulose membranes (Hybond N, GE Healthcare, Chicago, IL, USA). The membranes were blocked for up to 1 h using fat free milk in PBS (5% w/vol) and bovine serum albumin (2% w/vol) containing 0.1% Tween 20 (PBS-T). The blots were incubated for 1 h at room temperature (RT) with the appropriate primary antibody diluted in PBS-T, as described (31). The primary antibodies used were (i) mouse monoclonal antibodies (MAbs) to His_6_ (GE Healthcare, Chicago, IL, USA) diluted 1:1,000; (ii) MAb 2F2 (MRA-184) to PvCSP from the VK210 strain (1 µg/mL) (32); and (iii) MAb 2E10E9 (MRA-185) to PvCSP from the VK247 strain (1 µg/mL) developed by Dr. Alan Cochrane (Unpublished data). The hybridomas used for the production of MRA-184 and MRA-185 were obtained from the Malaria Research and Reference Reagent Resource Center (MR4), Manassas, VA, USA.

After washing twice with PBS-T, peroxidase-labeled goat anti-mouse IgG (Sigma-Aldrich, St. Louis, MO, USA) diluted 1:1,000 in PBS-T was added for 1 h. The reaction was developed using a chemiluminescence detection assay (ECL, GE Healthcare, Chicago, IL, USA).

#### Reverse-Phase High-Performance Liquid Chromatography (RP-HPLC)

Purified proteins were analyzed by RP-HPLC using a C18 column (4.6 × 250 mm; 300 µm particle size) coupled to an HPLC LCMS-2020 LC/MS system (Shimadzu, Kyoto, Japan). The HPLC procedure was performed at RT (≈25°C) using a binary gradient of 0.1% trifluoroacetic acid (TFA, Solvent A) and 0.1% TFA in a 9:1 (v/v) solution of acetonitrile:water (Solvent B) with a two-step solvent gradient starting at 0–20%, followed by 20–100%, at a rate of 1 mL/min for 40 min. The elution was monitored with a UV-Visible absorbance detector (Shimadzu SPD M20A) at 220 and 280 nm.

### Recombinant Proteins Expressed in *E. coli*

In order to determine the specificity of IgG antibodies, flagellin-fusion proteins His_6_FliC-PvCS-VK210 (31), His_6_FliC-PvCS-VK247 and His_6_FliC-PvCS-*P. vivax*-like (28), were used individually on ELISA plates for analyzing the specificity of the antibodies against each of the repeats sequences, as these proteins do not have the C-terminal region. The His_6_FliC protein (31), used as a carrier for the individual repeat sequences, is a recombinant flagellin derived from *Salmonella* Typhimurium and produced in *E. coli*. In order to determine the specificity of the antibodies against C-terminal region, No repeats protein (29) was used. This recombinant protein contains only the PvCSP N-terminal region fused with the C-terminal region.

These recombinant proteins were produced in *E. coli* using the method previously described (33). Following this, purification was performed by affinity chromatography (HisTrap FF column, GE Healthcare, Chicago, IL, USA) and subsequent anion-exchange chromatography (Resource Q column, GE Healthcare, Chicago, IL, USA) as described above.

### Immunization Schedule

For immunological assays, 6–8 weeks old C57BL/6 female mice were subcutaneously (s.c.) immunized thrice, following the schedule described in the figures (*n* = 6 mice per group). For each dose, the indicated amount of protein was administered with high-molecular-weight Poly(I:C) adjuvant (50 μg/dose/mouse) (InvivoGen, San Diego, CA, USA). A volume of 50 µL was injected s.c. into each footpad (first dose) and a final volume of 100 µL was injected s.c. at the base of the tail (second and third doses). After each immunization, blood was collected from the tail, and the sera were analyzed for the presence of antibodies against each recombinant protein. For the protection experiment, mice were intraperitoneally immunized thrice, following the schedule described in Figure 8A.

### Immunological Assays

#### Antibody Measurement

Antibodies against each recombinant protein in mouse sera were detected by enzyme-linked immunosorbent assay (ELISA), as described previously (29). The recombinant proteins were employed as solid phase-bound antigens (200 ng/well), and a volume of 50 µL of each solution was added to each well of a 96-well plate. After overnight incubation at RT, plates were washed with a solution of PBS and 0.05% PBS-T and blocked with a solution of PBS, 2.5% (w/v) skimmed milk, and 2.5% BSA for 2 h at 37°C. Serial dilutions (1/2) of murine polyclonal sera (100 µL) were added to wells in duplicate, followed by incubation for 1 h at 37°C. After washing with PBS-T, 50 µL aliquots of peroxidase-labeled goat anti-mouse IgG (Sigma, St. Louis, MO, USA), diluted 1:1,000, were added to each well and incubated for 1 h at 37°C. The enzymatic reaction was developed with o-phenylenediamine (1 mg/mL) (Sigma) diluted in phosphate-citrate buffer (pH 5.0) containing hydrogen peroxide [0.03% (vol/vol)]. The enzymatic reaction was stopped by the addition of 50 µL of a solution containing 4 N H_2_SO_4_. The optical density at 492 nm (OD_492_) was measured using a SpectraMax Plus 384 Microplate Reader. Anti-PvCSP titers were determined based on the highest dilution of sera that yielded an A_492_ higher than 0.1. This cut-off value was set at 3 SDs above the mean A_492_ obtained from naïve mice against the recombinant proteins.

For detection of IgG subclass responses, secondary antibodies specific to mouse IgG1, IgG2b, and IgG2c were used (Southern Technologies, Chattanooga, TN, USA). Results are expressed as the mean values of IgG titers ± SD.

#### Immunofluorescence Assay

*Anopheles cracens* mosquitoes were fed on human blood obtained from infected Thai patients using Hemotek^®^ membrane-feeding system (34). After 2 weeks, aseptically dissected infected salivary glands were disrupted in a glass tissue grinder and the sporozoite preparation was deposited on glass slides. Slides were dried at RT and kept frozen at −20°C before use.

Slides containing wild-type VK210 *P. vivax* sporozoites were fixed with cold methanol for 10 min and blocked with PBS–3% BSA for 30 min at RT. Slides were then incubated in a humid chamber for 1 h at RT with sera from mice immunized with the mix of the three CSP alleles (VK210, VK247, and *P. vivax*-like) or with the yPvCSP-All epitopes recombinant protein (dilution 1:100 in PBS–3% BSA). MAb 2F2 (anti-PvCSP-VK210) and sera from mice immunized with adjuvant only (dilution 1:50 in PBS–3% BSA) were used as a positive and negative controls, respectively. Slides were washed three times with PBS-T before the addition of a dilution 1:500 in PBS–3% BSA of anti-mouse IgG conjugated to Alexa Flour 568 (Thermo Fisher Scientific, Waltham, MA, USA). The slides were incubated for 1 h at RT, washed three times with PBS-T and incubated for 15 min with 4′,6-Diamidino-2-Phenylindole Dihydrochloride (DAPI, Invitrogen, Carlsbad, CA, USA). Binding was visualized using a fluorescence microscope DMI6000B/AF6000 (Leica) coupled to a digital camera system (DFC 365 FX) and the images were treated and analyzed using the open-source software ImageJ.

#### Cytokine Measurement

To measure the number of cells secreting interferon gamma (IFN-γ), splenocytes collected from immunized C57BL/6 mice were used for enzyme-linked immunospot (ELISPOT) assays. This procedure was performed by using mouse IFN-γ capture antibody (BD Biosciences) to coat flat-bottom Multiscreen HTS plates (Millipore) overnight at 4°C. After three washes with PBS-T, the plates were blocked with R10 [(fetal calf serum (10%) (v/v), RPMI 1640), Gibco], for 2 h at 37°C and 5 × 10^5^ cells/well mice splenocytes were stimulated overnight at 37°C, 5% CO_2_, in the presence of recombinant proteins (yPvCSP-VK210, yPvCSP-VK247, yPvCSP-*P. vivax*-like, and yPvCSP-All epitopes, 10 mg/mL) or ConA (2.5 mg/mL) diluted in RPMI 1640-IL2 [IL-2 0.03% (v/v)]. Plates were then washed 3 times with PBS, and mouse IFN-γ detection antibody biotinylated [XMG 1.2, (1:200), Pharmingen] was added to the plates overnight at 4°C. Streptavidin-labeled peroxidase [(1:500), BD, Biosciences] was added to the plates, after six PBS-T washes, for 2 h and spots were visualized with 4′,6-diamidino-2-phenylindole, dihydrochloride (1 mg/mL).

To determine the intracellular expression of IFN-γ and tumor necrosis factor alpha (TNF-α), intracellular cytokine staining (ICS) was used. These assays were performed as in previous studies (29, 35). Basically, ICS was evaluated following the *in vitro* culture of splenocytes in the presence or absence of an antigenic stimulus. Cells were washed three times in RPMI 1640 medium (pH 7.4) and resuspended in cell culture medium consisting of RPMI 1640 medium (pH 7.4) supplemented with 10 mM HEPES, 0.2% sodium bicarbonate, 59 mg/L of penicillin, 133 mg/L of streptomycin, and 10% fetal bovine serum (Hyclone, Logan, UT, USA). Cell viability was evaluated using 0.2% trypan blue exclusion dye. Cell density was adjusted to 5 × 10^6^ cells/mL in cell culture media containing anti-CD28 (2 µg/mL), BDGolgiPlug (10 µg/mL), monensin (5 µg/mL), and FITC-labeled anti-CD107a (2 µg/mL). Final concentrations of 10 µg/mL of the indicated recombinant proteins or 2 µg/mL of Concanavalin A (ConA; Sigma-Aldrich, St. Louis, MO, USA) were added. The cells were cultivated in V-bottom 96-well plates (Corning, New York, NY, USA) in a final volume of 200 µL in duplicate, at 37°C in a humid environment containing 5% CO_2_. After a 12 h incubation, cells were stained for surface markers with PerCP-Cy5.5-labeled anti-CD4 (clone RM4-5) or PECy7-labeled CD8 (clone 53-6.7) on ice for 20 min. To detect IFN-γ and TNF-α by intracellular staining, cells were then washed twice in PBS/0.5% BSA/2 mM EDTA, fixed and permeabilized with BD Fixation/Permeabilization solution. Cells were stained for intracellular markers using APC-labeled anti-IFN-γ (Clone XMG1.2) and PE-labeled anti-TNF-α (clone MP6-XT22). Finally, cells were washed twice with BD perm/wash buffer and fixed in 1% PBS-paraformaldehyde. At least 50,000 cells were acquired on a BD FACS Canto II flow cytometer and then analyzed with FlowJo 8.7 software. Gating strategy is depicted in Figure S1 in Supplementary Material.

### Mice Infection and Parasitemia Analysis

Sporozoites (spz) from *Plasmodium berghei* ANKA expressing *P. vivax* circumsporozoite VK210 repeats (Pb/PvVK210) were obtained as described elsewhere (36). Transgenic Pb/PvVK210 spz were maintained in female *Anopheles stephensi* mosquitoes. The total number of spz was determined using a Kova glass slide and 4,000 spz in 1 μL of PBS were microinjected s.c. in the footpad of C57BL/6 mice previously immunized as described above. Thin-blood smears were prepared daily from day 3 to day 10 after challenge, on glass slides from a drop of blood obtained from tails. Blood smears were air-dried, fixed with methanol, and Giemsa stained. Percentages of parasitized erythrocytes were determined by microscopic examination of ~3,000 erythrocytes each in Giemsa-stained smears.

For analysis of the hepatic infection, livers of immunized mice were harvested 42 h after sporozoite challenge and frozen in liquid nitrogen. Ground tissue was resuspended in Trizol reagent (Invitrogen) and total RNA was extracted according to the manufacturer’s instructions. After treatment with turboDNase (Ambion), 2 µg of total RNA was used for cDNA synthesis, using the superscript II reverse transcriptase and random primers (d(N)9, New England Biolabs). For each sample, a reaction without reverse transcriptase was used for controlling DNA contamination. The real time PCR was performed with 1/20 of the cDNA reaction using the iTaq Universal SYBR Green Supermix (Biorad) and the primers for the mouse HPRT (Fwd, 5′-CCTGCTGGATTACATTAAAGCACTG-3′; Rev5′-GTCAAGGGCATATCCAACAAC-3′) or for the *P. berghei* 18S rRNA (Fwd, 5′-AAGCATTAAATAAAGCGAATACATCCTTAC-3′; Rev, 5′-GGAGATTGGTTTTGACGTTTATGTG-3′). Reactions were run in triplicate in three independent experiments according to the following conditions: (1 × 95°C for 5 min; 40 × 95°C for 15 s, 60°C for 30 s; 1 × 55°C to 95°C in 13 min).

### Statistical Analysis

For immunological responses analyses, values were log-transformed and compared using one-way ANOVA followed by Tukey’s HSD tests.^3^ For parasitemia analyses, the non-parametric Mann–Whitney test was performed. Differences were considered statistically significant when *P* < 0.05.

## Results

### Four Recombinant Proteins Based on the Sequences of *P. vivax* CSPs Were Successfully Produced in the Yeast *P. pastoris* as Soluble Secreted Proteins

**Figure 2:**
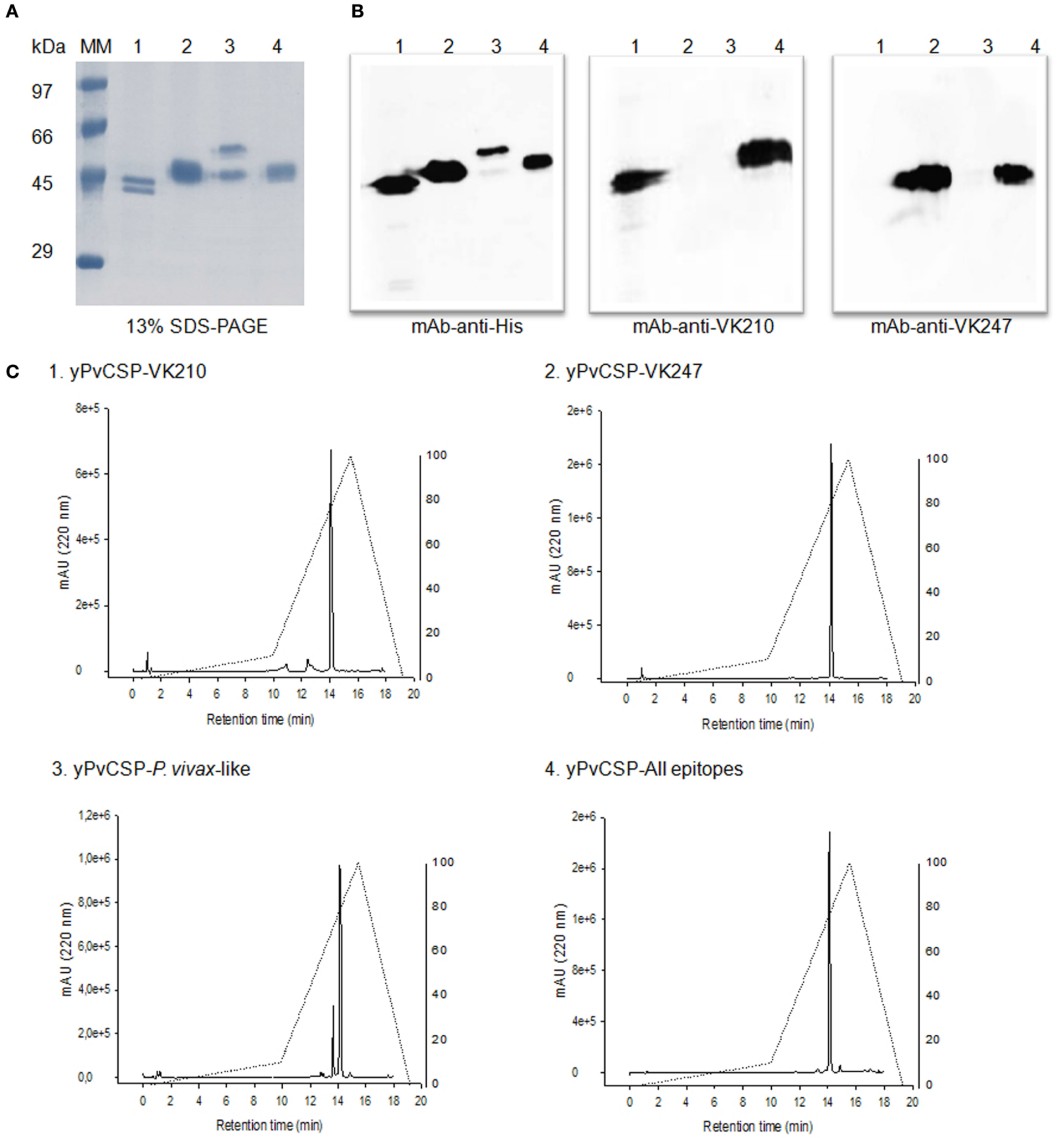
Purification and biochemical characterization of the *Plasmodium vivax* circumsporozoite protein (CSP) recombinant proteins. **(A)** 13% SDS-PAGE under reduced conditions stained with Coomassie blue. Proteins migrate between 50 and 55 kDa. Fractions were 2 µg of: (1) yPvCSP-VK210, (2) yPvCSP-VK247, (3) yPvCSP-*P. vivax*-like, and (4) yPvCSP-All epitopes. **(B)** Western blot using 1 µg of the same fractions of proteins described above. Antibodies used were monoclonal antibody (MAb) anti-His, MAb VK210 (2F2), and MAb VK247 (2E10.E9). The secondary antibody used was anti-mouse IgG HRP-labeled, and detection was performed by ECL assay. **(C)** The purity of proteins, after the combination of chromatographic methods, was analyzed by reverse-phase high-performance liquid chromatography (RP-HPLC), in which the gradient elution was developed combining 0.1% trifluoroacetic acid (TFA) in water and 0.1% TFA in 90% acetonitrile, 24°C, 1 mL/min for 40 min, in a C18 column.

Recombinant proteins representing the PvCSP alleles (VK210, VK247, and *P. vivax*-like) as well as the hybrid polypeptide yPvCSP-All epitopes were generated. This hybrid contained the conserved C-terminal of the *P. vivax* CSP and the three variant repeat domains in tandem. The proteins were purified and their integrity and purity was evaluated by SDS-PAGE, immunoblotting, and RP-HPLC (Figure 2). A reduced gel stained with Coomassie blue showed that the proteins migrated between 40 and 55 kDa (Figure 2A). Figure 2B shows that the purified proteins were recognized by specific MAbs generated against radiation-attenuated *P. vivax* sporozoites (MAb VK210 2F2 and MAb VK247 2E10.E9), as well as monoclonal anti-His antibody. Recognition was also observed for the entire hybrid polypeptide. The purity of the proteins, after the combination of chromatographic methods, was analyzed by RP-HPLC. Analysis of RP-HPLC chromatograms revealed high purity of these recombinant proteins (Figure 2C).

### Vaccine Containing the Three Allelic Forms of the *P. vivax* CSP Was Immunogenic in Mice

**Figure 3:**
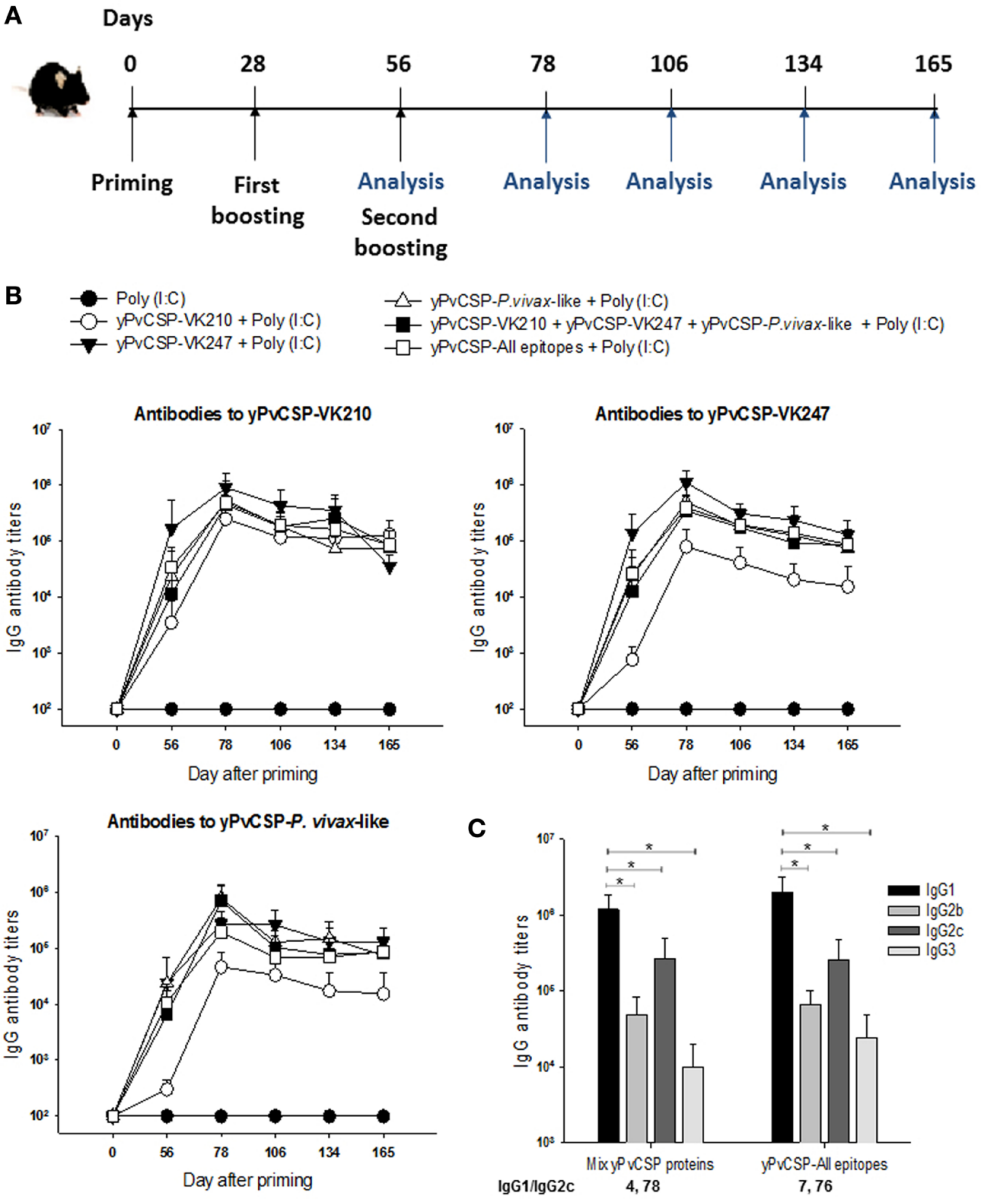
Specific antibody response in mice immunized with *Plasmodium vivax* circumsporozoite protein (PvCSP) recombinants. **(A)** C57BL/6 mice were immunized s.c. with three doses 28 days apart with the purified proteins in the presence of the adjuvant Poly(I:C), whereby the antibody response was analyzed according to the timeline described. **(B)** The IgG antibody serum titers against recombinant proteins representing each allelic form of PvCSP were analyzed by ELISA. The results are expressed as the means ± SD (*n* = 6). Mouse groups were immunized with: (1) Poly(I:C); (2) Poly(I:C) plus yPvCSP-VK210 (1 μg/dose/mouse); (3) Poly(I:C) plus yPvCSP-VK247 (1 μg/dose/mouse); (4) Poly(I:C) plus yPvCSP-*P. vivax*-like (1 μg/dose/mouse); (5) Poly(I:C) plus a protein mix [yPvCSP-VK210 (1 μg/dose/mouse) + yPvCSP-VK247 (1 μg/dose/mouse) + yPvCSP-*P. vivax*-like (1 μg/dose/mouse)]; or (6) Poly(I:C) plus yPvCSP-All epitopes (3 μg/dose/mouse). **(C)** IgG isotypes were determined by ELISA in sera of mice from groups 5 and 6 described above. Asterisk denotes statistical differences (*P* < 0.01) when comparing IgG1 isotype with IgG2b, IgG2c and IgG3.

The serum IgG responses to PvCSP antigens were determined in C57BL/6 mice immunized s.c. with the purified proteins in the presence of the adjuvant Poly(I:C). Six mice per group were immunized with three doses containing 1 µg of each protein or adjuvant alone administered as described in Section “Materials and Methods,” at 28-day intervals. Formulations containing the mix of the three CSP alleles (VK210, VK247, and *P. vivax*-like, 1 µg of each protein/animal/dose) or the hybrid polypeptide (yPvCSP-All epitopes, 3 µg of protein/animal/dose) were also tested. The antibody titers were analyzed by ELISA according to the timeline described in Figure 3A. These homologous prime-boost vaccination regimens were highly immunogenic in the mouse model, eliciting a high and long-lasting specific antibody immune response (Figure 3B).

To better characterize the anti-PvCSP response, the IgG subtypes of the generated antibodies were analyzed and the IgG1/IgG2c ratio was calculated. All mouse sera presented detectable levels of all IgGs in the groups immunized with the proteins individually, mixed, or fused. An analysis of the IgG1/IgG2c anti-PvCSP-VK210 ratio (Figure 3C), showed a polarized Th2-like response (IgG1/IgG2c > 1). Similar results were observed against PvCSP-VK247 (data not shown).

**Figure 4:**
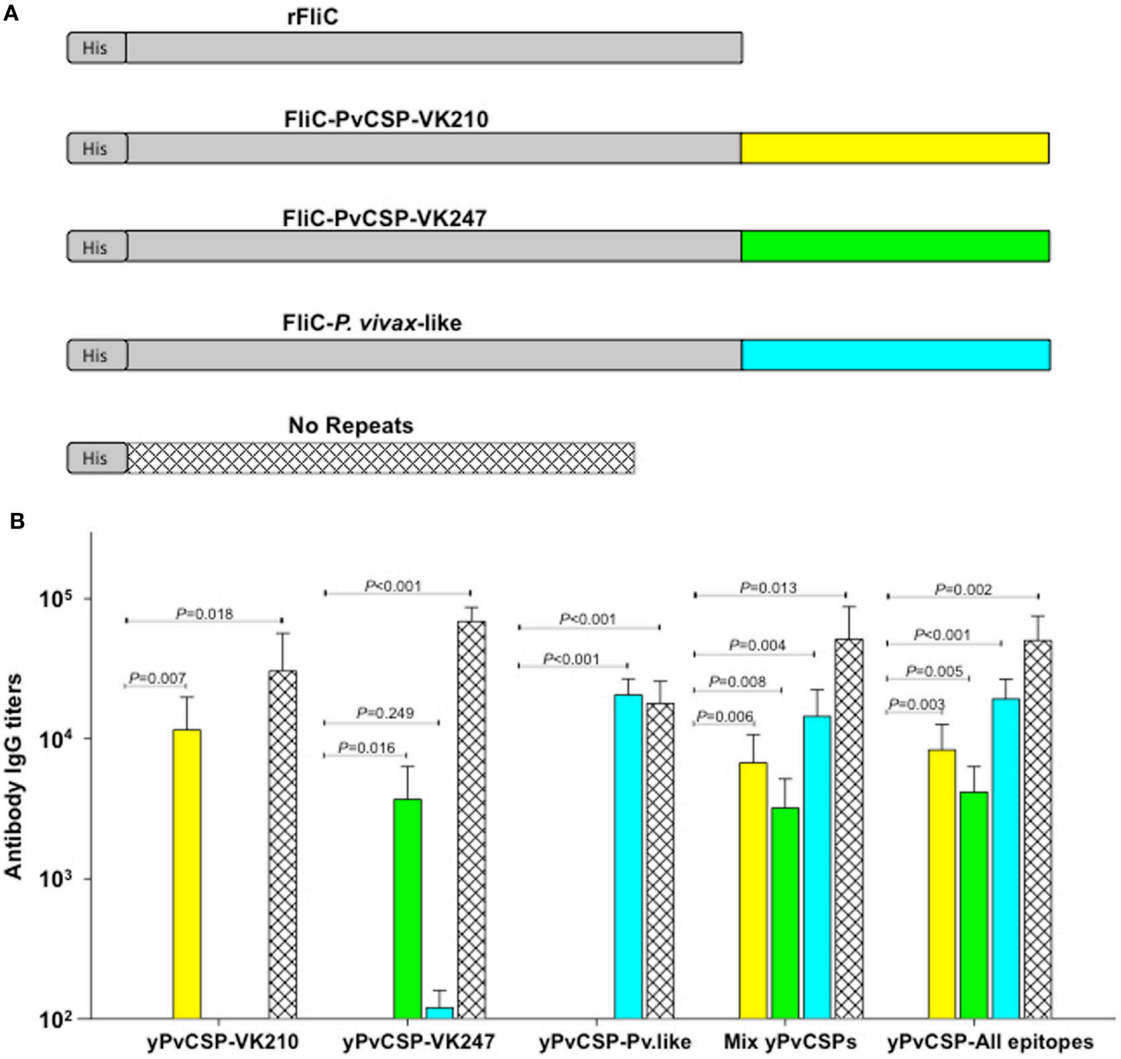
Specificity of IgG antibody in mice immunized with *Plasmodium vivax* circumsporozoite protein (CSP) recombinants. **(A)** Schematic representation of recombinant proteins used as a substrate bound to the plates in the ELISA assay. **(B)** C57BL/6 mice were immunized s.c. with the purified proteins in the presence of the adjuvant Poly(I:C) according to the timeline described in Figure 3A. The specificity of IgG antibody serum titers toward rFliC, FliC-PvCSP-repeats and No repeats proteins were analyzed by ELISA in sera of mice from groups 2 to 6 described in Figure 3B. The results are expressed as mean ± SD (*n* = 6 animals/group). *P* values are indicated. Differences were considered statistically significant when *P* < 0.05.

To delineate the specificity of anti-yPvCSP-All epitopes antibodies, when compared with immunizations performed with individual yPvCSP proteins or protein mix, the mouse serum was tested against three recombinant proteins containing only the repeats fused to flagellin (FliC) of *Salmonella enterica* serovar Typhimurium [FliC-PvCSP-repeats (28)], and one recombinant protein containing only PvCSP N- and C-terminal regions [No repeats (29)] (Figure 4A). Interestingly, all FliC-PvCSP-repeats variants and the No-repeats protein were recognized in sera from mice immunized with protein mix or with yPvCSP-All epitopes, whereas no significative cross-reaction among repeat sequences was observed in animals immunized with individual yPvCSP proteins (Figure 4B). We concluded that the immunization with a recombinant protein expressing all the three different allelic variants in fusion elicited high IgG antibody titers reacting with all three different allelic variants of PvCSP. The antibodies were targeted to both the C-terminal and the repeat domains of PvCSP.

**Figure 5:**
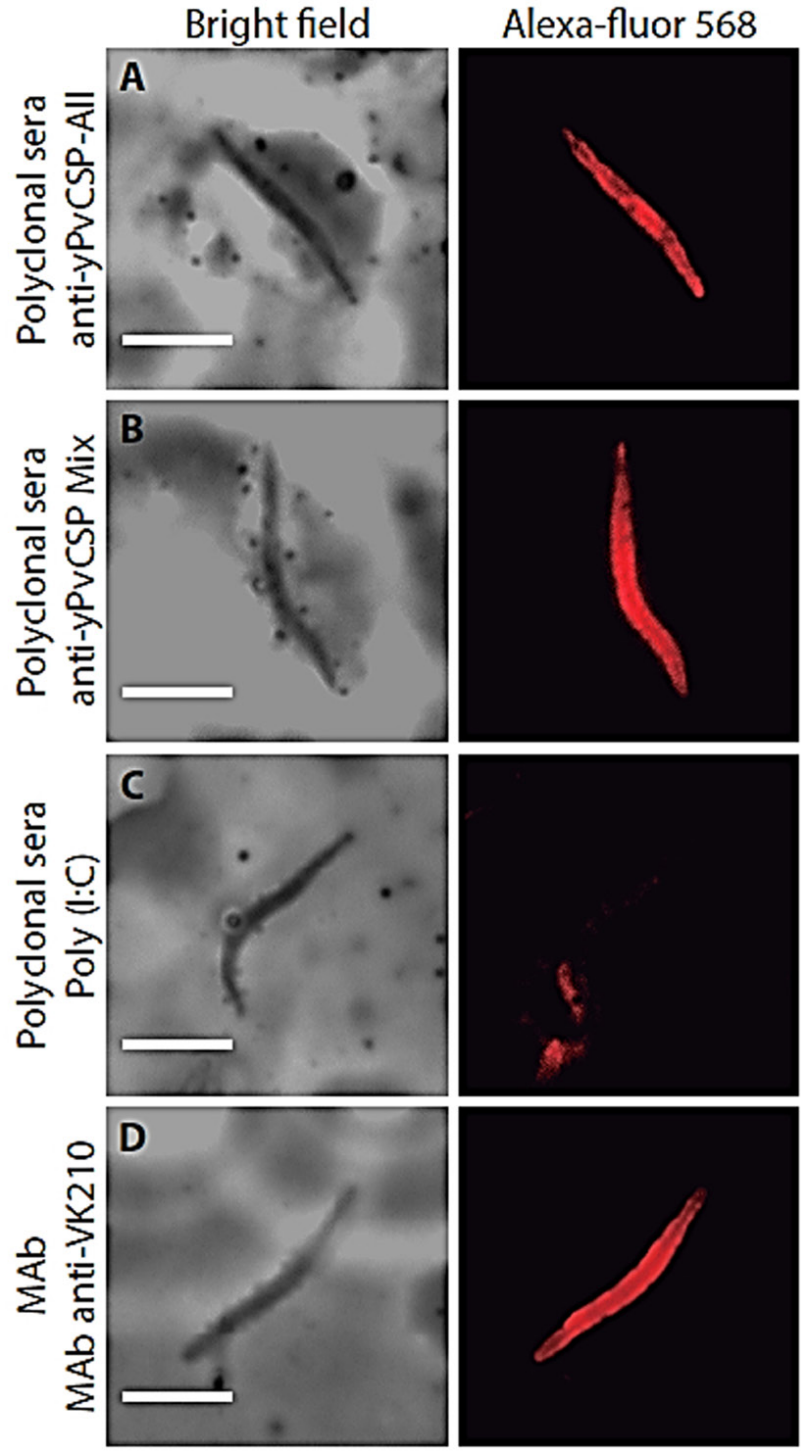
Recognition of native protein in *Plasmodium vivax* isolates. Indirect immunofluorescence analysis was performed using pool of sera (diluted 1:100) from C57BL/6 mice immunized with **(A)** yPvCSP-All epitopes, **(B)** protein mixture or **(C)** phosphate-buffered saline (PBS) in adjuvant, as a negative control. **(D)** As a control positive anti-PvCSP-VK210 (MAb 2F2) was used. Microscope slides containing wild-type *P. vivax* were obtained from Thailand isolates. Antibody binding was detected with secondary Alexa 568-labeled antibody (red) and nuclei were visualized by DAPI staining.

In addition, we determined whether sera from mice immunized with the prime-boost immunization regimen reacted with *P. vivax* sporozoites in immunofluorescence assays. We observed that sera from both groups of mice that were immunized with a formulation containing the yPvCSP-All epitopes and protein mixture reacted to sporozoites of the *P. vivax* VK210 strain (Figures 5A,B). Antibody recognition was specific, as control sera from mice immunized with the adjuvant Poly(I:C) did not react (Figure 5C), and the sera-stained sporozoites showed a pattern of staining on the surface of the sporozoites similar to that observed with the monoclonal antibody anti-VK210 (Figure 5D).

### yPvCSP-All Epitopes and Protein Mix Induced Low Activation of CD4^+^ and CD8^+^ T Cells

**Figure 6:**
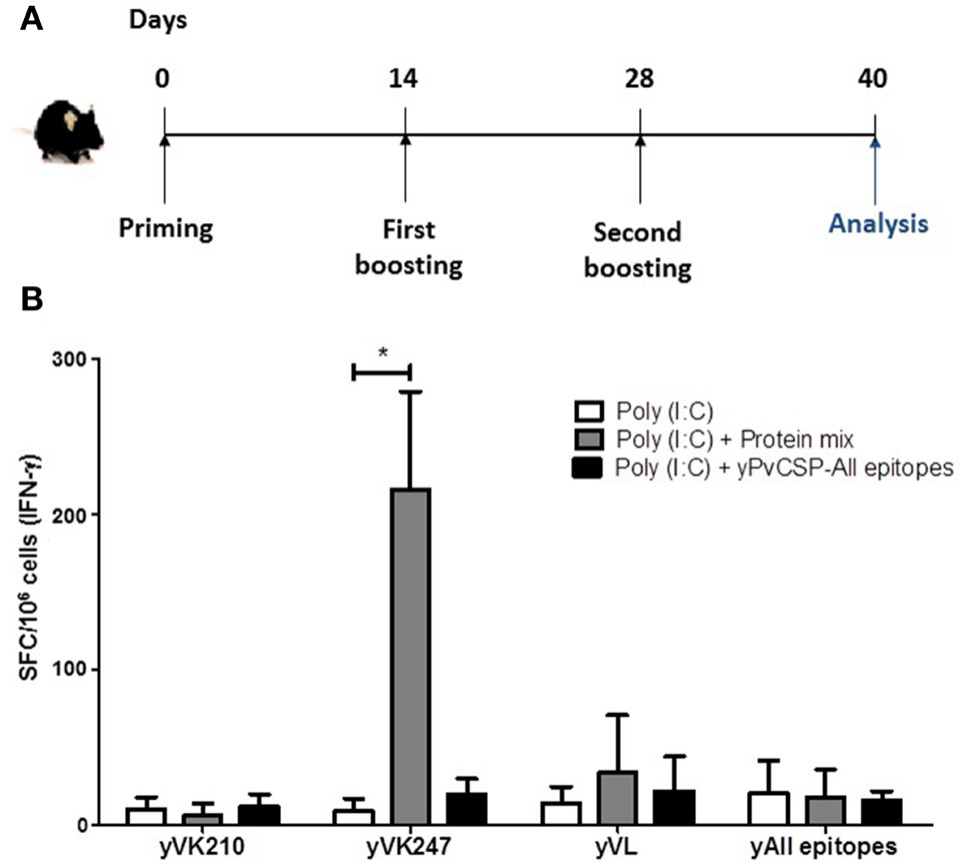
Interferon gamma (IFN-γ)-producing cells in mice immunized with recombinant proteins. **(A)** C57BL/6 mice were immunized following the scheme with yPvCSP-All epitopes (3 μg/mouse) or a mix of yPvCSP-VK210 + yPvCSP-VK247 + yPvCSP-*P. vivax*-like (1 μg/each protein/mouse), in the presence of the adjuvant Poly(I:C). **(B)** To measure the number of cells secreting IFN-γ, splenocytes collected from immunized C57BL/6 mice on day 40 after priming were used for enzyme-linked immunospot (ELISPOT) assays. The recombinant proteins yPvCSP-VK210 (yVK210), yPvCSP-VK247 (yVK247), yPvCSP-*P. vivax*-like (yVL), and yPvCS-All epitopes (yAll epitopes) were used as stimuli (10 µg/mL). SFC, spot forming cells. Asterisk denotes statistical differences (*P* < 0.05).

We selected two vaccine formulations (yPvCSP-All epitopes and protein mix) to evaluate the ability of the induction of the T cell-mediated immune responses in immunized C57BL/6 mice. We measured IFN-γ secretion by ELISPOT assay. We also evaluated the production of inflammatory cytokines (IFN-γ and TNF-α) by CD4^+^ and CD8^+^ T cells using ICS. Figure 6A shows a schematic representation of the immunization schedule. Spleen cells were stimulated *in vitro* with recombinant proteins representing the three allelic forms of PvCSP.

Overall, very low levels of CD4^+^ and CD8^+^ T cells producing any cytokine were detected. Positive controls performed in parallel with stimulation with ConA were consistently successful (data not shown). A higher number of IFN-γ-producing cells were observed in the group immunized with the protein mix and stimulated with recombinant yPvCSP-VK247, but not in the animals immunized with the yPvCSP-All epitopes when the splenocytes were stimulated with the same protein (Figure 6B). This difference is in agreement with previous results (29) and could be due to a different processing or antigen presentation of the VK247 epitope.

**Figure 7:**
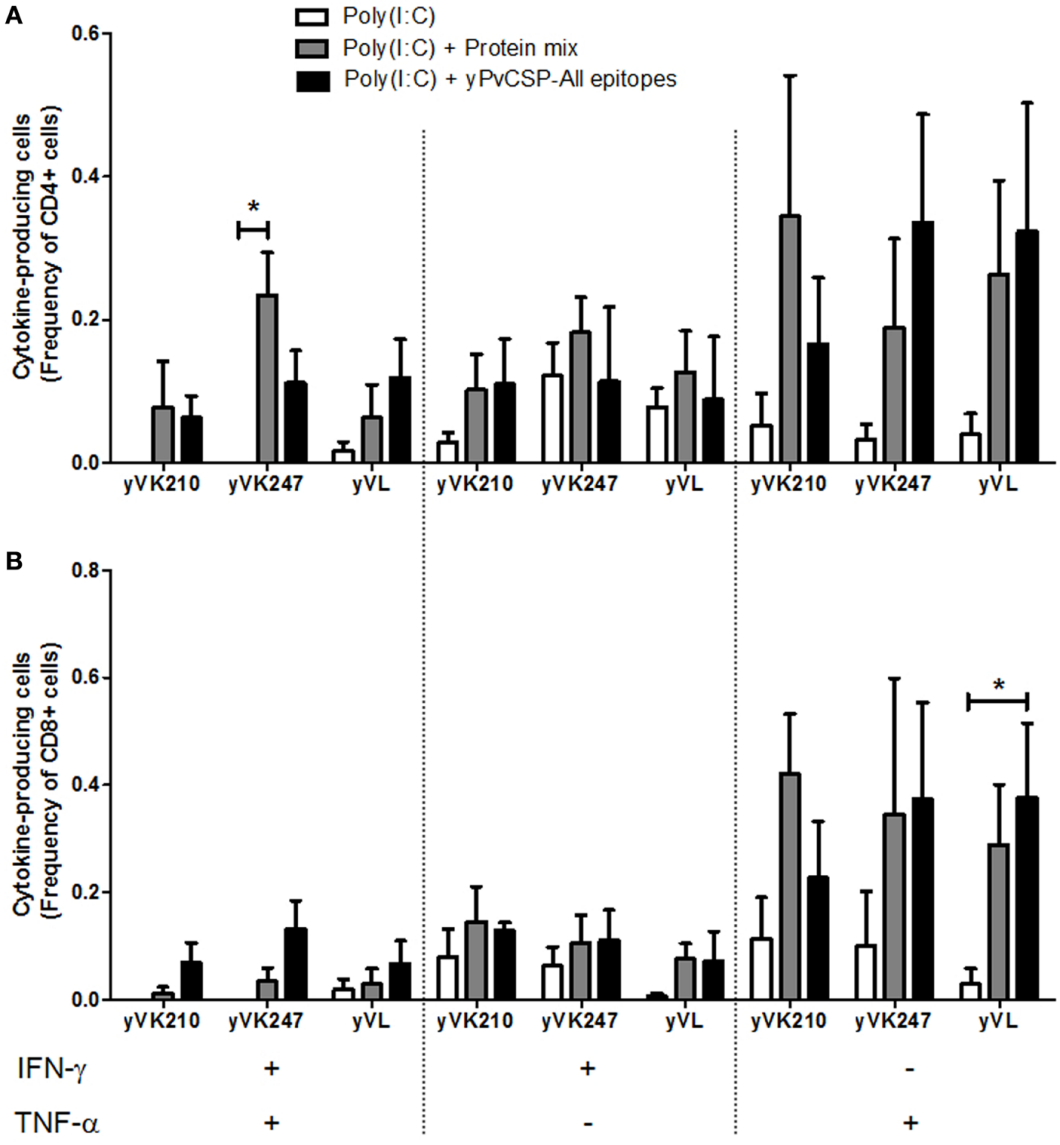
CD4^+^ and CD8^+^ T cell-mediated immunity of mice immunized with recombinant proteins. C57BL/6 mice were immunized s.c. with the recombinant proteins following the scheme described in Figure 6A. To determine the intracellular expression of interferon gamma (IFN-γ) and tumor necrosis factor alpha (TNF-α), intracellular cytokine staining (ICS) was performed following the *in vitro* culture of splenocytes in the presence or absence of the antigenic stimuli yPvCSP-VK210 (yVK210), yPvCSP-VK247 (yVK247), and yPvCSP-*P. vivax*-like (yVL). **(A)** Frequency of CD4^+^ cytokine-producing cells. **(B)** Frequency of CD8^+^ cytokine-producing cells. Asterisks denote statistically significant (*P* < 0.05) higher frequencies of cells from mice immunized with the recombinant proteins when compared to cells from mice injected with adjuvant only. Media backgrounds of all samples were subtracted before plotting.

We also attempted to detect IFN-γ and TNF-α using ICS (Figure 7). Using Boolean gating analysis, we were able to detect low but significant simultaneous production of both cytokines by CD4^+^ T cells derived from mice immunized with the protein mix in a splenocyte culture restimulated with yPvCSP-VK247 recombinant protein (Figure 7A). We also observed that CD8^+^ T cells derived from mice immunized with the protein yPvCSP-All epitopes were able to produce low but significant levels of TNF-α in a splenocyte culture pulsed with the antigen yPvCSP-*P. vivax*-like (Figure 7B). On the other hand, we did not detect any specific responses (IFN-γ or TNF-α) in CD4^+^ or CD8^+^ T cells after restimulation with recombinant yPvCSP-VK210.

Similarly, we attempt to detect a cytotoxic response in vaccinated mice by measuring CD107a expression in CD4^+^ or CD8^+^ T cells after stimulation with the recombinant proteins. Under the experimental conditions, we did not detect significant differences in CD107a expression when compared to mice immunized with adjuvant only (Figure S2 in Supplementary Material).

### A Vaccine Containing the Three Allelic Forms of the *P. vivax* CSP Induces Protection against Infection in Mice after Challenge with Chimeric Parasite Pb/PvVK210

**Figure 8:**
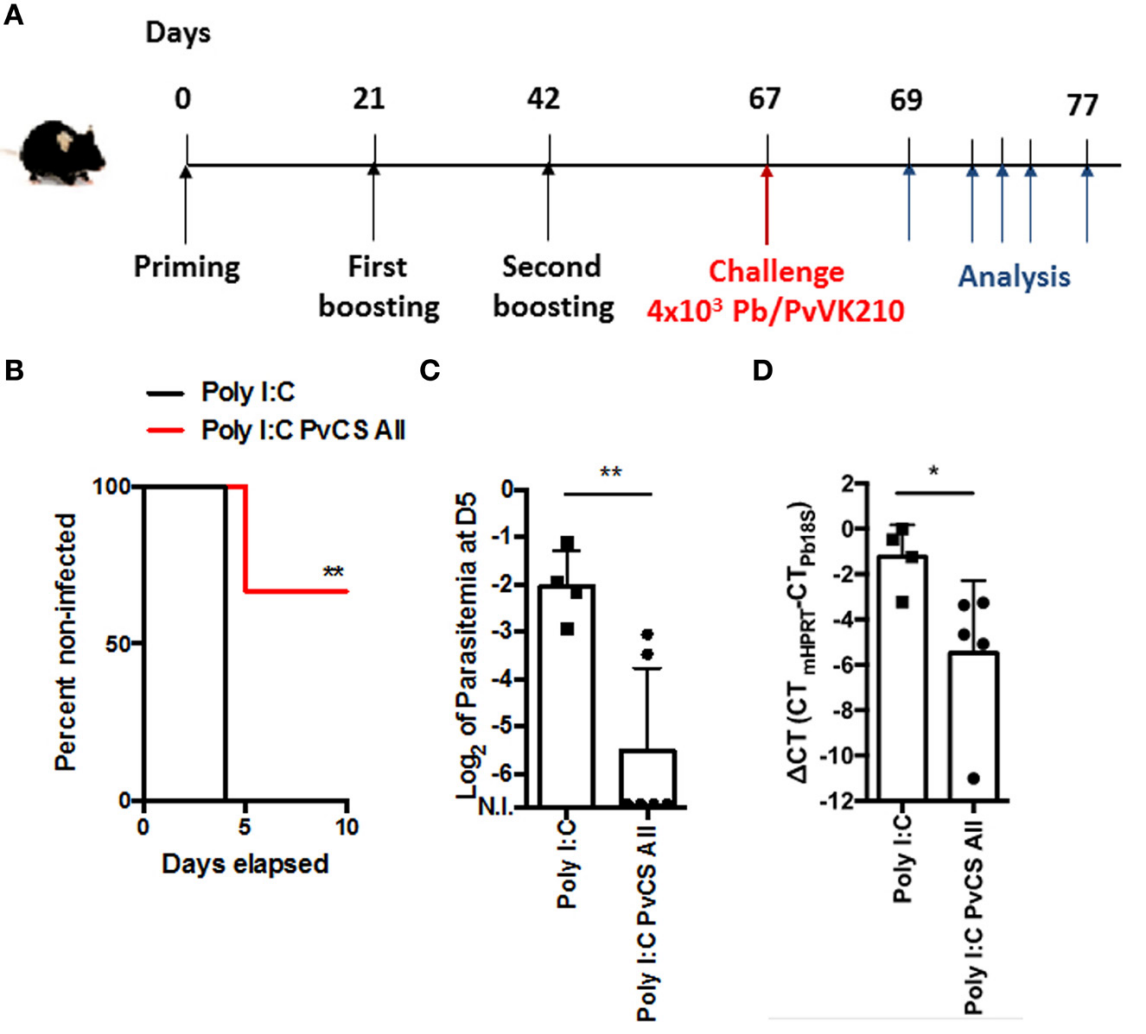
Protection against experimental challenge in mice immunized with a prime-boost regimen. **(A)** C57BL/6 mice were immunized i.p. with Poly(I:C) or a mixture of Poly(I:C) plus 5 µg of yPvCSP-All epitopes following the scheme described. On day 67 after priming, mice were challenged with 4,000 Pb/PvVK210 transgenic sporozoites. **(B)** Parasitemia was analyzed by Giemsa-stained blood smears on the following 10 days. Percentage of non-infected mice in each experimental group are shown. **(C)** Log of parasitemia at day 5 (D5) postchallenge measured in mice immunized with Poly(I:C) (*n* = 4) or a mixture of Poly(I:C) plus 5 µg of yPvCSP-All epitopes (*n* = 6). (**) *P* = 0.0095. **(D)** The liver load was evaluated by RT-PCR. The infection level in liver in mice immunized with a mixture of Poly(I:C) plus 5 µg of yPvCSP-All epitopes (*n* = 5) is compared with control group that received Poly(I:C) alone (*n* = 4). (*) *P* = 0.0159. C-terminal (CT) differences (delta CT) between the mouse HPRT and Pb 18S CT values are shown.

We used the chimeric parasite Pb/PvVK210, in which the repeats of the PbCSP were replaced by those of the PvCSP (VK210), to evaluate the potential efficacy of the yPvCSP-All epitopes vaccine against a sporozoite infection *in vivo*. C57BL/6 mice were immunized with yPvCSP-All epitopes in the presence of Poly(I:C), in a three-dose prime-boost immunization regimen (Figure 8A). The immunization-challenge schedule is described in Figure 8A.

This experimental design involved challenge with 4,000 Pb/PvVK210 sporozoites on day 25 after boost. As shown in Figure 8B, the immunization with three doses of 5 µg of protein/mouse/dose was protective for 4/6 mice until day 10 postchallenge, whereas all control mice became infected by day 4 postchallenge. This protection corresponded to a ~10-fold decrease in parasitemia at day 5, when infected blood cells in mice that only received adjuvant still increase exponentially (Figure 8C, *P* = *0*.0095).

Finally, we evaluated the liver stage parasite burden in immunized mice challenged with Pb/PvVK210 sporozoites. The liver parasite burdens in mice group immunized with a mixture of Poly(I:C) plus 5 µg of yPvCSP-All epitopes was 20 times lower compared with control group that received Poly(I:C) alone (*P* = *0*.0159) (Figure 8D).

## Discussion

In the past decade, diverse efforts toward the development of an effective vaccine against malaria have increased the knowledge about the level of protection necessary—although also often not enough—to achieve disease-preventative immunity. For preerythrocytic vaccines, there is a consensus on the requirement of high serum levels of IgG antibodies specific to the repeat sequences of the CSP antigen, both for *P. falciparum* and for *P. vivax*. In addition, protection has been related to the frequency of CD4^+^ T cells expressing proinflammatory cytokines; thus, several approaches have been trialed in order to produce both a strong antibody response and cell-mediated immunity.

In several studies focusing on RTS,S formulations, the generation of antibodies against the central repeat region of *P. falciparum* CSP has been recognized as key factor to achieve some level of protective effect [reviewed in Ref. (11)]. Accordingly, ELISA titers to the CSP repeats are significantly higher in protected individuals, whereas opsonizing antibodies would not contribute to protection and the C-terminal-targeted antibody contribution remains undefined (37).

In *P. vivax*, the development of a vaccine targeting the CSP antigen has been limited for several decades to preclinical immunization models developed mostly in mice. In previous works, we were able to successfully elicit high titers of antibodies recognizing the three allelic forms of the PvCSP antigen, by using homologous or heterologous prime-boost immunization regimens (29). These immunizations were carried out using the same adjuvant employed here, Poly(I:C), which is a synthetic analog of double-stranded RNA capable of activating toll-like receptor type 3 and therefore eliciting a virus-like immune response in humans (38). However, the recombinant protein expression from the bacterial system yielded high levels of unfolded protein, mostly recovered from inclusion bodies, which have to be denatured to obtain purified proteins. These production difficulties led us to develop novel CSP-based recombinant proteins to be expressed in a eukaryotic system such as *P. pastoris*. Our group has been employing this protein-production system successfully for the last few years, enabling the easy production of highly purified, stable, properly folded, and immunogenic proteins to be used for the development of vaccines against protozoa parasites (30, 39). These features, also shown in this work, would be a great advantage for the following scaling process in order to obtain a vaccine formulation, which could be distributed in several endemic areas.

Recently, results from the first in-human clinical trial aiming to develop a vaccine against *P. vivax* (VMP001) were published. This formulation contains a recombinant protein comprising N-terminal, C-terminal, VK210, and VK247 repeat domains. As mentioned, vaccination did not induce sterile protection; however, a significant delay in the prepatent period was associated with the presence of anti-VK210 repeats-specific responses (27).

Interestingly, although specific antibodies were generated against all regions of the CSP in all vaccinated individuals, CD4^+^ T cell responses specific to repeat and C-terminal regions were detected in only 17% of vaccinated subjects, whereas 90% of subjects showed strong cellular responses to the N-terminal region (27). As this response was not protective against natural challenge (i.e., infected-mosquito bites), we think that immunodominant epitopes of the *P. vivax* CSP-N-terminal region could be hampering the recognition or antigen presentation of repeat-specific domains. Therefore, the absence of the N-terminal region in our formulations, which were able to induce a repeat-specific antibody and cellular responses in mice, could present an advantage when compared to VMP001 formulations.

To our knowledge, this is the first report of a vaccine potentially protective against all the three allelic variants of *P. vivax* CSP. The results presented herein show that immunization with our recombinant proteins was able to induce a strong humoral immune response, eliciting Abs against all three repeat regions as well against C-terminal region. Although immunization with single variants generated antibodies with some level of cross-reaction between the different variants of PvCSP, we showed in previous studies that the cross-reactivity between IgG antibodies is very limited and would not be possible to use a single variant as a universal vaccine (28). Furthermore, cross-reactivity is expected to be due essentially to Abs recognizing the C-terminal region, present in all the proteins. As Abs against the repeat regions of PvCSP were demonstrated to be associated to protection in *A. nancymaae* monkeys (14), the vaccine formulation containing the hybrid polypeptide (yPvCS-All epitopes) was selected for protection assays, aiming at further development of an anti-*P. vivax* vaccine. The relevant proportion of the antibody titer specific for each of the repeat regions induced by immunization with yPvCSP-All epitopes indicates that one (instead of three) stable protein could be able to confer protection against all the three allelic variants of *P. vivax* sporozoites.

The precise reason why the All epitopes vaccine elicited a very low cytokine response measured in the ICS assay and an undetectable response in the ELISpot assay is not clear to date. A challenging objective of future works will be to find adjuvants, delivering systems or expression strategies able to improve CSP-specific cellular responses. Combined use of viral vectors and potent adjuvants was recently reported to improve CD8^+^ T cell responses against preerythrocytic malaria (40). We are currently working on some of these strategies using new adjuvants, adenovirus-delivered antigens and Virus-like particles formulations in order to further develop cell-mediated responses.

In summary, the strong humoral response showed in this work, with a significant (>10^4^) proportion of antibodies targeting repeats sequences, and the low but detectable CD4^+^ and CD8^+^ T cell-mediated responses, indicate that this formulation should be highly immunogenic in humans, generating immunity to all three variants of PvCSP. Furthermore, the significant protection elicited after challenge with 4,000 Pb/PvVK210 sporozoites should indicate a more effective protection against natural infection, in which ~100 sporozoites are released per bite (41).

Although the protection found here was not sterilizing to all animals, it has been hypothesized that a candidate *P. vivax* vaccine with low efficacy against primary infection may potentially reduce transmission by preventing relapses (42). In addition, still it is possible to improve the protective immunity of the recombinant vaccine yPvCSP-All epitopes combining with other potent adjuvants (Adjuvant Systems) (43). Taken together, our results are encouraging for testing our vaccine formulations in clinical trials, aiming to the development of a multi-allelic vaccine against *P. vivax* malaria.

## Ethics Statement

This study was performed in strict accordance with the recommendations in the Guide for the Care and Use of Laboratory Animals of the Brazilian National Council for the Control of Animal Experimentation (CONCEA) (http://www.mctic.gov.br/mctic/opencms/textogeral/concea.html). The protocol was approved by the Committees on the Ethics of Animal Experiments of the Faculty of Pharmaceutical Sciences of University of Sao Paulo, Brazil (CEUA No. 362/2012), and the Institutional Animal Care and Use Committee at the Federal University of Sao Paulo (CEP No. 0172/12 and CEUA No. 1463171214). The study with human blood obtained from infected Thai patients was approved by Oxford Tropical Research Ethics Committee (reference OX28-09).

## Author Contributions

Designed the study and associated protocols: MR, RA, and IS. Performed research work: AG, LL, KF, PD, RP, and J-MT. Analyzed data: AG, LL, KF, DB, MR, RA, and IS. Contributed reagents and materials: CA, FN, LR, RN, and VN. Wrote the manuscript: AG, RA, and IS. All authors read and approved the final version of the manuscript.

## Conflict of Interest Statement

The authors declare that the research was conducted in the absence of any commercial or financial relationships that could be construed as a potential conflict of interest.

## Footnotes

^1^https://clinicaltrials.gov.

^2^http://www.mctic.gov.br/mctic/opencms/textogeral/concea.html.

^3^http://faculty.vassar.edu/lowry/VassarStats.html.
