# Supplementary figures and images for: Vaccine Containing the Three Allelic Variants of the Plasmodium vivax Circumsporozoite Antigen Induces Protection in Mice after Challenge with a Transgenic Rodent Malaria Parasite
Source: Front Immunol. 2017 Oct 11;8:1275. doi: 10.3389/fimmu.2017.01275 (PMC5642139; doi:10.3389/fimmu.2017.01275)

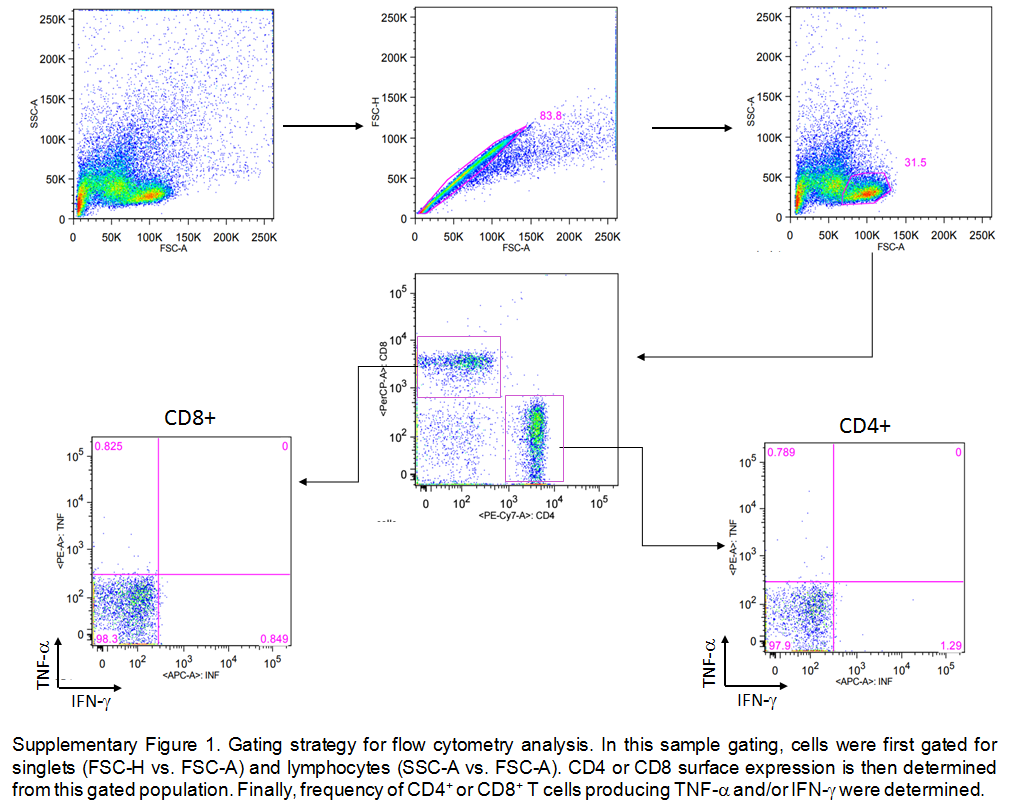

Supplement: Supplementary file 1 [file Image_1.TIFF]

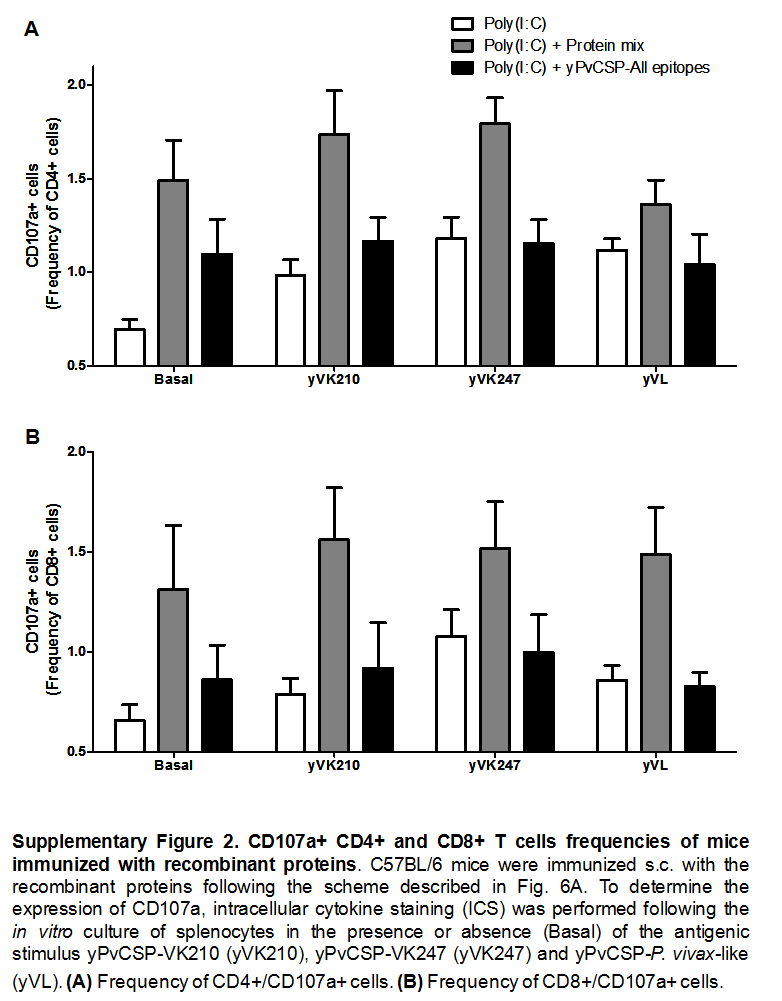

Supplement: Supplementary file 2 [file Image_2.TIFF]
